# Effects of Androgen Deprivation on Cerebral Morphometry in Prostate Cancer Patients – An Exploratory Study

**DOI:** 10.1371/journal.pone.0072032

**Published:** 2013-08-19

**Authors:** Herta H. Chao, Sien Hu, Jaime S. Ide, Edward Uchio, Sheng Zhang, Michal Rose, John Concato, Chiang-shan R. Li

**Affiliations:** 1 Department of Internal Medicine & Yale Comprehensive Cancer Center, Yale University School of Medicine, New Haven, Connecticut, United States of America; 2 Medical Service, VA Connecticut Healthcare System, West Haven, Connecticut, United States of America; 3 Department of Psychiatry, Yale University School of Medicine, New Haven, Connecticut, United States of America; 4 Department of Science and Technology, Federal University of Sao Paolo, Salo Paolo, Brazil; 5 Tower Research, Los Angeles, California, United States of America; 6 Clinical Epidemiology Research Center, VA Connecticut Healthcare System, West Haven, Connecticut, United States of America; 7 Deparment of Neurobiology, Yale University School of Medicine, New Haven, Connecticut, United States of America; 8 Interdepartmental Neuroscience Program, Yale University, New Haven, Connecticut, United States of America; University of North Carolina at Chapel Hill, United States of America

## Abstract

**Background:**

Androgen deprivation therapy (ADT) is a common treatment for non-metastatic, low-risk prostate cancer, but a potential side effect of ADT is impaired brain functioning. Previous work with functional magnetic resonance imaging (MRI) demonstrated altered prefrontal cortical activations in cognitive control, with undetectable changes in behavioral performance. Given the utility of brain imaging in identifying the potentially deleterious effects of ADT on brain functions, the current study examined the effects of ADT on cerebral structures using high resolution MRI and voxel-based morphometry (VBM).

**Methods:**

High resolution T1 weighted image of the whole brain were acquired at baseline and six months after ADT for 12 prostate cancer patients and 12 demographically matched non-exposed control participants imaged at the same time points. Brain images were segmented into gray matter, white matter and cerebral ventricles using the VBM toolbox as implemented in Statistical Parametric Mapping 8.

**Results:**

Compared to baseline scan, prostate cancer patients undergoing ADT showed decreased gray matter volume in frontopolar cortex, dorsolateral prefrontal cortex and primary motor cortex, whereas the non-exposed control participants did not show such changes. In addition, the decrease in gray matter volume of the primary motor cortex showed a significant correlation with longer reaction time to target detection in a working memory task.

**Conclusions:**

ADT can affect cerebral gray matter volumes in prostate cancer patients. If replicated, these results may facilitate future studies of cognitive function and quality of life in men receiving ADT, and can also help clinicians weigh the benefits and risks of hormonal therapy in the treatment of prostate cancer.

## Introduction

Prostate cancer is the most common non-skin cancer in American men. Almost half of all patients with prostate cancer will receive androgen deprivation therapy (ADT) over the course of their illness [1], [2]. Although ADT has documented efficacy in the treatment of metastatic prostate cancer, ADT is also given to patients with non-metastatic prostate cancer, as neoadjuvant and adjuvant treatment and in patients who have a rising PSA level after definitive therapy. Evidence suggests that ADT can improve the survival of patients with non-metastatic prostate cancer with poor prognostic features, but no consensus exists regarding indications for or duration of ADT in the patients with lower-risk disease [3]. Despite this uncertainty, use of ADT has become more prevalent in the U.S. in recent years, accounting for most of price-adjusted growth in Medicare spending related to prostate cancer [4].

Common side effects of ADT include fatigue, decreased sexual function, gynecomastia, osteoporosis, and changes in metabolism, and the negative impact of these adverse effects on quality of life has been studied extensively [5]. It remains unclear, however, whether or to what extent ADT influences cognitive function, a major determinant of quality of life in these mostly elderly patients. Both observational and randomized studies support an association between androgens and cognitive function [6]–[10], and studies have suggested a protective effect of testosterone against age-related decline in cognitive function, including the development of dementia in men. Conversely, the effects of ADT on cognitive function in patients with prostate cancer remain unclear, with reports suggesting no effect [11], impaired function [12]–[14], and a mixed effect with patients showing an improved performance on some tests and a deterioration on others [15].

Magnetic resonance imaging (MRI) provides a non-invasive method to measure brain structures and activations. Investigators have used MRI and other imaging methods to evaluate the effects of chemotherapy and hormonal therapy on brain structures and functions in patients with breast cancer [16]–[19]. Prior work of functional MRI showed that compared to participants who did not receive ADT, prostate cancer patients undergoing ADT did not differ in cognitive performance, but they showed altered prefrontal cortical activation during cognitive control [20]. These and other findings suggested the utility of brain imaging to detect the effects of hormonal therapy on brain structures and functions before the manifestation of cognitive and behavioral effects.

In this context, the current study uses structural MRI to evaluate changes in cerebral gray matters among prostate cancer patients who receive ADT, compared with patients who do not receive the treatment.

## Materials and Methods

### Participants and Clinical Profiles

Patients were recruited from the Medical Oncology and Urology Clinics at the VA Connecticut Healthcare System. Potential candidates were identified at the bi-monthly Genito-Urinary Tumor Board, or during their routine clinic visits, and offered to participate in the study. Eighteen men who had non-metastatic, biopsy proven prostate cancer and who were prescribed ADT–either as adjuvant treatment or because of biochemical recurrence after prostatectomy or radiotherapy–were recruited to participate in the study. ADT consisted of medical castration with a luteinizing hormone releasing hormone agonist (Goserelin 10.8 mg subcutaneously every 90 days) after a lead-in period for 2 weeks with bicalutamide 50 mg daily. During the same time interval, 18 age-matched individuals with non-metastatic prostate cancer who were undergoing radiotherapy or had prostatectomy and had never been treated with ADT were enrolled as non-exposed control participants. Exclusion criteria included: currently active second malignancy; Eastern Cooperative Oncology Group (ECOG) Performance Status >1; any significant cardiovascular conditions (e.g., New York Heart Association (NYHA) Class III or IV congestive heart failure, recent myocardial infarction, unstable angina, pacemaker); or hepatic (e.g., liver cirrhosis Child-Pugh B or C), renal, or neurological disease. Patients who had a diagnosis of axis-I psychiatric or substance (excluding nicotine) use disorders [21], and patients receiving any investigational agents, were excluded. A score of less than 27 out of 30 on the mini-mental state examination (MMSE) was another exclusion criterion [22]. All participants underwent a health questionnaire interview to ensure eligibility for fMRI prior to the study.

Written informed consent was obtained in accordance to institutional guidelines and procedures approved by the Yale Human Investigation Committee and the Human Investigation Subcommittee of the Veterans Affairs (VA) Connecticut Health Care System. Of the original study population, 12 ADT and 12 control participants completed the structural brain scans both at baseline and 6 months (after treatment) (**Table 1**).

**Table 1:** Patient characteristics and quality of life rating.

Group	ADT; n = 12	Control; n = 12
Age	69.1±5.6 years	65.5±6.6 years
Education	9^th^ grade: 1	9^th^ grade: 1
	High School/GED: 3	High School/GED: 3
	College 1–3 years: 3	College 1–3 years: 2
	College graduate: 2	College graduate: 3
	Post-graduate: 3	Post-graduate: 3
MMSE	29.1±1.1	29.5±0.7
Cancer staging	Stage 1: 0	Stage 1: 1
	Stage 2: 10	Stage 2: 9
	Stage 3: 2	Stage 3: 2
Local therapy	Radiation 100%	Radiation 33.3%
	Surgery 0%	Surgery 58.3%
	Surgery+Radiation 0%	Surgery+Radiation 8.3%
QOL at baseline	116±25	128±18
QOL of lifeat 6 months	111±23	124±21
Testosteroneat 6 months	0.16±0.11 ng/ml	2.88±1.01 ng/ml

Note: ADT: Androgen Deprivation Therapy; GED: General Education Development Test; MMSE: Mini Mental State Examination; QOL: FACT-P quality of life score; there is no difference between groups in age (t = 2.080, p = 0.166, two-sided two-sample t test); there is no difference between groups in MMSE score (t = 2.101, p = 0.273, two-sided two-sample t test); Staging follows the current guidelines of the 2010 American Joint Committee on Cancer (AJCC); there is also no difference across the two time points in the change of QOL between groups (F = 0.015, p = 0.905, interaction, repeated measures analysis of variance).

### Working Memory: N-back Task

Working memory is a form of short-term memory that allows individuals to hold and manipulate information in mind in order to do complex tasks such as comprehension, reasoning, and learning. A behavioral paradigm widely used to study working memory is the N-back task. In the N-back task, participants view a series of letters and respond to a letter (a target) that matches the previous letter (“1-back”) or the letter two time steps back (“2-back”). Thus, to detect the target, participants need to hold the memory of the letters that appear consecutively for a period of time. In “0-back” trials, participants simply respond to a pre-designated target letter. Zero-, 1- and 2- back trials are run in different blocks and both the accuracy rate (percentage of the target letters correctly identified) and the reaction time (RT) of correct trials are used as indices of performance. A higher accuracy rate and shorter RT represents better working memory.

Our participants performed an N-back working memory task outside the scanner at baseline and 6 months following treatment. Participants responded to a series of letters presented at a rate of 1 every 2 s (stimulus duration = 500 ms). Fifteen phonologically distinct letters served as stimuli (A, B, C, D, E, F, G, H, K, M, N, P, S, W, X), with three blocks in the task, differing in working memory load. Each subject performed 3 sessions of the N-back task, with each session comprising two each of 0-, 1-, and 2- back blocks, the order of which was counter-balanced across sessions. Each block began with an information screen showing the “load” for that block (5 s) and contained 24 trials, with one-third of them representing targets. Correct response rate and reaction time of correct trials were recorded for each block and averaged for the each load condition for analyses.

### Subjective Assessment of Quality of Life

As a general assessment of overall status, participants completed standardized Quality-of-Life-Questionnaires (QOL) for prostate cancer patients (Fact-P©) at baseline and again at 6 months [23].

### Imaging Protocol

Participants were scanned on a Siemens 3-Tesla scanner (Trio; Siemens AG, Erlangen, Germany). Data for each participant consisted of a single high-resolution T1-weighted gradient-echo scan: 176 slices; 1 mm^3^ isotropic voxels; field of view = 256×256 mm; data acquisition matrix = 256×256; TR = 2530 ms; TE = 3.66 ms, bandwidth = 181 Hz/pixel; flip angle = 7°.

### Voxel-based Morphometry (VBM)

The aim of VBM is to identify differences in the local composition of brain tissue and its association with behavioral and cognitive measures, while discounting large scale differences in gross anatomy and position. This can be achieved by spatially normalizing individuals’ structural images to the same stereotactic space, segmenting the normalized images into distinct brain tissues, smoothing the gray-matter images, and performing a statistical test to localize significant associations between anatomical and behavioral measures [24].

Voxel-based morphometry was performed using the VBM8 toolbox (http://dbm.neuro.uni-jena.de/vbm/) packaged in Statistical Parametric Mapping 8 (Wellcome Department of Imaging Neuroscience, University College London, U.K.). T_1_-images were first co-registered to the Montreal Neurological Institute or MNI template space (1.5 mm^3^ isotropic voxels) using a multiple stage affine transformation, during which the 12 parameters of interest were estimated. Co-registration started with a coarse affine registration using mean square differences, followed by a fine affine registration using mutual information. In this step, coefficients of the basis functions that minimize the residual square difference (between individual image and the template) were estimated. Tissue probability maps constructed from 471 healthy subjects were used in affine transformation. After affine transformation, T_1_-images were corrected for intensity bias field (kernel size FWHM = 60 mm) and a local means denoising filter [25] with default parameter 1 was applied, to account for intensity variations (inhomogeneity) and noise caused by different positions of cranial structures within MRI coil. The images were then segmented into cerebrospinal fluid, gray and white-matters, using an adaptive maximum a posteriori method [26] with k-means initializations, as implemented in VBM8, generating tissue class (including gray matter or GM) maps.

In segmentation, partial volume estimation was performed with default parameter 5, with a simplified mixed model of at most two tissue types [27]. Segmented and the initially registered tissue class maps were normalized using Dartel [28], a fast diffeomorphic image registration algorithm of SPM. As a high-dimensional non-linear spatial normalization method, Dartel generates mathematically consistent inverse spatial transformations. We used the standard Dartel template in MNI space, constructed from 550 healthy subjects of the IXI-database (http://www.brain-development.org/), to drive the Dartel normalization. Normalized GM maps were modulated to obtain the absolute volume of GM tissue corrected for individual brain sizes. Finally, the GM maps were smoothed by convolving with an isotropic Gaussian kernel. Smoothing helps to compensate for the inexact nature of spatial normalization and reduces the number of statistical comparisons (thus making the correction for multiple comparisons less severe); however, it reduces the accuracy of localization. Most VBM studies used a kernel size of FWHM = 12 mm. We used a smaller kernel size of FWHM = 8 mm to achieve localization accuracy.

In group analyses, we compared the cerebral gray matter volume at baseline and after treatment, for both the ADT and non-exposed group, using paired-sample t test. We also derived the gray matter volumes of the regions of interest for individual participants and correlated these measurements with changes in performance on the working memory task.

## Results

### Quality of Life Scores

No statistically significant differences between ADT and control patients were found in QOL scores at baseline, or with regard to change over 6 months using the FACT-P^©^ questionnaire (**Table 1**).

### Performance in N-back Task

**As shown in** **Table 2**, the results of N-back task performance scores indicated that the correct response rate decreased with increasing memory load for both ADT and non-exposed participants. We conducted two-way repeated measures ANOVA separately for 0-, 1-, and 2- back data. The results indicated indistinguishable N-back task performance between the two groups across the two time points.

**Table 2:** Performance in the N-back working memory task.

	0-back (correct %)	0-back (RT, ms)	1-back (correct %)	1-back (RT, ms)	2-back (correct %)	2-back (RT, ms)
ADT_B	98±3	593±116	82±13	669±153	62±16	765±153
ADT_F	97±5	569±103	85±12	720±181	64±18	805±189
Control_B	99±1	494±89	89±12	575±109	77±15	719±138
Control_F	99±2	563±70	88±4	659±125	82±9	745±116
P value*	0.79	0.19	0.50	0.19	0.55	0.78

Note: B: baseline; F: follow-up; RT = reaction time of correct trials;

* P value of the group by time interaction in repeated measures analysis of variance.

### Voxel-based Morphometry

**Figure 1:**
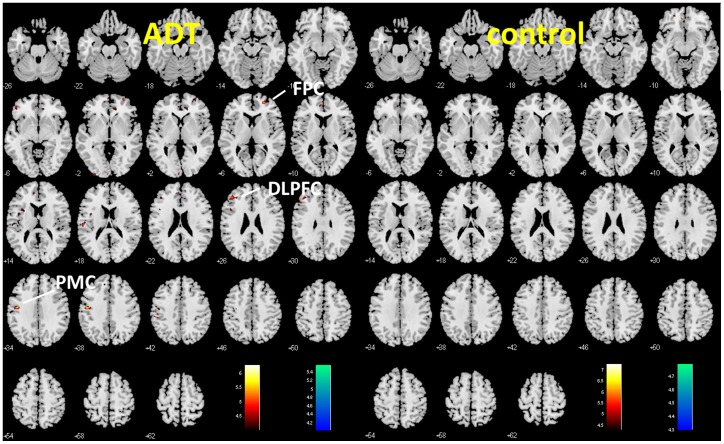
Changes in cerebral gray matter volumes in frontal cortices as demonstrated by voxel-based morphometry. Voxelwise paired t test between baseline and 6 months for ADT (patients who received 6 months of androgen deprivation therapy) and Control (patients who did not receive any hormonal therapy) group, at p<0.001, uncorrected. The difference in gray matter volume, as reflected by a map of T values (color bar), is shown here on a structural brain image in axial sections, from z = -26 to z = +62, with adjacent sections 4 mm apart. Warm color: baseline >6 months; Cool color: 6 months>baseline. Neurological orientation: Right (R) = right. ADT but not control patients showed decreased gray matter volume in the primary motor cortex (PMC), frontopolar cortex (FPC), and dorsolateral prefrontal cortex (DLPFC).

Paired sample t-tests were used for voxel-wise comparison between gray matter volumes at the two time points, for both the ADT and control group. The results showed decreased gray matter volumes for the ADT group but not the control group in the primary motor cortex (x = −48, y = −10, z = 37, 185 voxels, Z = 4.02), frontopolar cortex (x = 23, y = 51, z = 4, 158 voxels, Z = 3.91), and dorsolateral prefrontal cortex (x = −42, y = 38, z = 27, 192 voxels, Z = 3.67), p<0.001, uncorrected, as shown in **Figure 1**. A flexible factorial analysis of variance with time point as a within-subject factor confirmed that the gray matter volumes in the primary motor and dorsolateral prefrontal cortices were significantly lower 6 months after ADT, when compared to baseline, in the ADT group in contrast with the control group (p<0.05, small volume correction for family-wise error of multiple comparisons).

**Figure 2:**
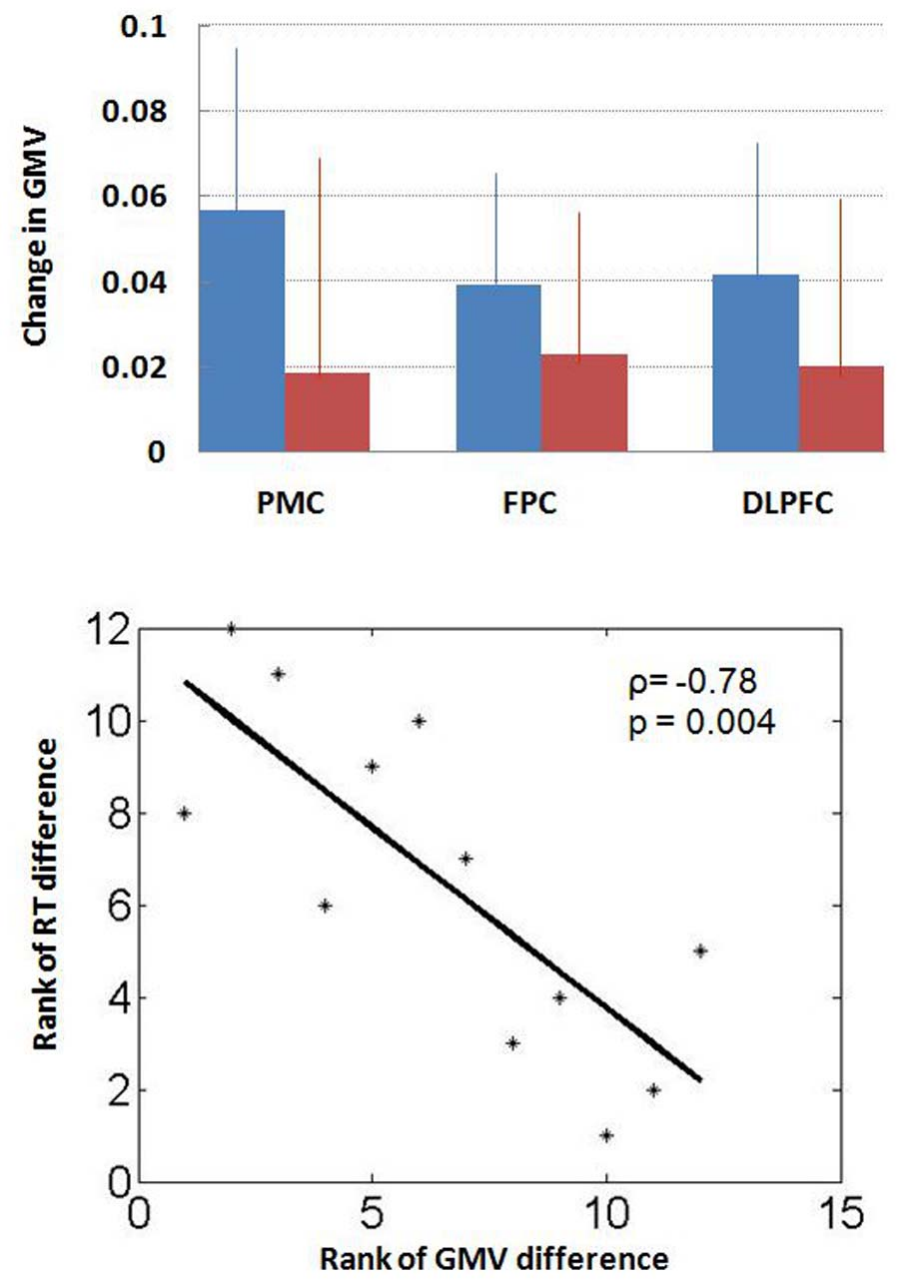
Changes (mean ± s.d.) in Gray matter volume (GMV) for the three regions of interest: primary motor cortex (PMC), frontopolar cortex (FPC), and dorsolateral prefrontal cortex (DLPFC), for the ADT (blue) and control (red) group (upper panel). A decrease in the GMV of the PMC correlated with prolonged reaction time to target detection during the zero-back condition in the ADT group (lower panel). Because of the small sample size, we used a Spearman regression for correlation (p<0.0042, rho = −0.7832). The result of Pearson regression was also significant: p<0.0049.

We extracted the gray matter volume of these two regions of interest for all patients and correlated these changes in cerebral gray matter volume to changes in performance in the N-back task (**Figure 2** **upper panel**). The results showed that a decrease in the gray matter volume of the primary motor cortex correlated with prolonged reaction time to target detection during the zero-back condition in the ADT group (p<0.0042, rho = −0.7832, Spearman regression, **Figure 2** **lower panel**). The gray matter change of the primary motor cortex and dorsolateral prefrontal cortex did not correlate with the change in accuracy or reaction time in the one- or two- back condition, for the ADT or control group (all p’s >0.107).

## Discussion

Studies of traditional neurocognitive testing without brain imaging have shown variable effects of ADT on cognitive function. In the current study, we observed a decrease in gray matter volumes in frontal and prefrontal cortical structures associated with the use of ADT. The decrease in gray matter volume of the primary motor cortex correlated with increased response time – suggesting processing insufficiency – during target detection in the N-back task. These results are consistent with our previous study that showed ADT-induced altered medial prefrontal cortical activation during cognitive control [20], and with the study by Cherrier and colleagues, which showed ADT-induced changes in parieto-occipital activation during spatial memory [29]. Our patients were matched by age and level of education, and all assessments were performed either before or at least 3 months after any surgery or radiation treatment to minimize any impact of treatment-related symptoms. Together, these results suggest that androgen deprivation may have a deleterious impact on cerebral structures and functions that are not evident using traditional behavioral tests [30].

Previous studies have shown that regional sex differences in gray matter volume are associated with sex hormones in the developing human brain [31]. Corrected for age, global gray matter volume was negatively associated with estradiol levels in girls, and positively with testosterone levels in boys [32]. Cerebral morphometric analysis suggested that gray matter development in certain brain regions is associated with sexual maturation and that pubertal hormones might have organizational effects on the developing human brain [33]. Children with Klinefelter syndrome who did not receive testosterone replacement therapy showed decreased total gray and white matter volumes [34]. Adults with Klinefelter syndrome also had a significant reduction of left temporal lobe volume and increased ventricular volume, which was inversely correlated with cognitive function [35]. In contrast, boys with familial male precocious puberty and early excessive androgen secretion were characterized by increased gray matter volume in several cortical and subcortical structures [36].

Data in adults also support the modulatory effects of androgen on cerebral cortical activities [37]–[41] and gray and white matter volume [34], [42]. A higher level of free testosterone was associated with greater cerebral blood flow in the hippocampus and prefrontal cortices in elderly men [37], and testosterone replacement therapy increased cerebral blood perfusion in the midbrain and prefrontal cortex in hypogonadal men [37]. In healthy women, administration of testosterone increased ventral striatal responses to reward [39].

Neuroimaging studies have reported differences in regional brain activations in neurological or psychiatric patients even when they performed at a level equal to their demographically-matched control participants. We argued earlier that these performance-independent changes in brain activities cannot be accounted for by effort or motivation, and potentially represent a correlate specific to the cerebral pathologies [20]. Here we demonstrate that androgen deprivation also alters cerebral morphometry. Since motivation or effort is highly unlikely to change cerebral morphometry within the time frame of the current experiment, these results provide additional evidence for potential side effects of ADT on the central nervous system. Nonetheless, we did not observe differences in the subjective report of quality of life or behavioral performance during the working memory task. More studies are thus required to thoroughly evaluate the overt impact of these functional and structural changes of the brain as a result of ADT.

A few limitations of our study need to be considered. First, the study involved a small sample size of 12 ADT and 12 control participants; thus, the results should be considered preliminary with the need for replication in future work. Second, the duration of observation is relatively short in the current cohort. Future studies should address whether the functional and structural differences associated with ADT worsen over time.

### Conclusion

Androgen deprivation for 6 months leads to structural brain changes in prostate cancer patients. Such changes are associated with prolonged reaction time to target detection in an N-back working memory task. The clinical implications of these changes are not known and warrant future study.

## References

[1] Meng MV, Grossfeld GD, Sadetsky N, Mehta SS, Lubeck DP, et al (2002) Contemporary patterns of androgen deprivation therapy use for newly diagnosed prostate cancer. Urology 60 (Suppl 3A)7–12. 10.1016/s0090-4295(02)01560-1 12231037

[2] Shahinian VB, Kuo Y-F, Freeman JL, Orihuela E, Goodwin JS (2005) Increasing use of gonadotropin-releasing hormone agonists for localized prostate cancer. Cancer 103: 1615–24. 15742331 10.1002/cncr.20955

[3] Dal Pra A, Cury FL, Souhami L (2010) Combining radiation therapy and androgen deprivation for localized prostate cancer-a critical review. Curr Oncol 17: 28–38. 10.3747/co.v17i5.632 PMC2949366 20975876

[4] Zhang Y, Skolarus TA, Miller DC, Wei JT, Hollenbeck BK (2011) Understanding prostate cancer spending growth among medicare beneficiaries. Urology 77: 326–331. 21168902 10.1016/j.urology.2010.09.025

[5] Alibhai SM, Gogov S, Allibhai Z (2006) Long-term side effects of androgen deprivation therapy in men with non-metastatic prostate cancer: a systematic literature review. Crit Rev Oncol Hematol 60: 201–215. 16860998 10.1016/j.critrevonc.2006.06.006

[6] Cherrier MM, Matsumoto AM, Amory JK, Johnson M, Craft S, et al (2007) Characterization of verbal and spatial memory changes from moderate to supraphysiological increases in serum testosterone in healthy older men. Psychoneuroendocrinology 32: 72–79. 17145137 10.1016/j.psyneuen.2006.10.008 PMC1864939

[7] Cherrier MM, Matsumoto AM, Amory JK, Asthana S, Bremner W, et al (2005) Testosterone improves spatial memory in men with Alzheimer disease and mild cognitive impairment. Neurology 64: 2063–2068. 15985573 10.1212/01.WNL.0000165995.98986.F1

[8] Kenny AM, Fabregas G, Song C, Biskup B, Bellantonio S (2004) Effects of testosterone on behavior, depression, and cognitive function in older men with mild cognitive loss. Journals of Gerontology. Series A, Biol Sci Med Sci 59: 75–78. 10.1093/gerona/59.1.m75 14718489

[9] Lu PH, Masterman DA, Mulnard R, Cotman C, Miller B, et al (2006) Effects of testosterone on cognition and mood in male patients with mild Alzheimer disease and healthy elderly men. Archives of Neurology 63: 177–185. 16344336 10.1001/archneur.63.2.nct50002

[10] Moffat SD, Zonderman AB, Metter EJ, Kawas C, Blackman MR, et al (2004) Free testosterone and risk for Alzheimer disease in older men. Neurology 62: 188–193. 14745052 10.1212/wnl.62.2.188

[11] Joly F, Alibhai SM, Galica J, Park A, Yi QL, et al (2006) Impact of androgen deprivation therapy on physical and cognitive function, as well as quality of life of patients with nonmetastatic prostate cancer. J Urol 176: 2443–2447. 17085125 10.1016/j.juro.2006.07.151

[12] Almeida OP, Waterreus A, Spry N, Flicker L, Martins RN (2004) One year follow-up study of the association between chemical castration, sex hormones, beta-amyloid, memory and depression in men. Psychoneuroendocrinology 29: 1071–1081. 15219659 10.1016/j.psyneuen.2003.11.002

[13] Green HJ, Pakenham KI, Headley BC, Yaxley J, Nicol DL, et al (2002) Altered cognitive function in men treated for prostate cancer with luteinizing hormone-releasing hormone analogues and cyproterone acetate: a randomized controlled trial.BJU Int. 90: 427–432. 10.1046/j.1464-410x.2002.02917.x 12175403

[14] Jenkins VA, Bloomfield DJ, Shilling VM, Edginton TL (2005) Does neoadjuvant hormone therapy for early prostate cancer affect cognition? Results from a pilot study. BJU Int. 96: 48–53. 10.1111/j.1464-410X.2005.05565.x 15963119

[15] Salminen E, Portin R, Korpela J, Backman H, Parvinen LM, et al (2003) Androgen deprivation and cognition in prostate cancer, Br J Cancer. 89: 971–976. 10.1038/sj.bjc.6601235 PMC2376935 12966411

[16] Castellon SA, Silverman DH, Ganz PA (2005) Breast cancer treatment and cognitive functioning: current status and future challenges in assessment. Breast Cancer Res Treat 92: 199–206. 16155790 10.1007/s10549-005-5342-0

[17] Eberling JL, Wu C, Tong-Turnbeaugh R, Jagust WJ (2004) Estrogen- and tamoxifen-associated effects on brain structure and function. Neuroimage 21: 364–371. 14741674 10.1016/j.neuroimage.2003.08.037

[18] Ferguson RJ, McDonald BC, Saykin AJ, Ahles TA (2007) Brain structure and function differences in monozygotic twins: possible effects of breast cancer chemotherapy. J Clin Oncol. 25: 3866–70. 10.1200/JCO.2007.10.8639 PMC3329758 17761972

[19] Silverman DH, Dy CJ, Castellon SA, Lai J, Pio BS, et al (2007) Altered frontocortical, cerebellar, and basal ganglia activity in adjuvant-treated breast cancer survivors 5–10 years after chemotherapy. Breast Cancer Res Treat. 103: 303–11. 10.1007/s10549-006-9380-z 17009108

[20] Chao HH, Uchio E, Zhang S, Hu S, Bednarski SR, et al (2012) Effects of androgen deprivation on brain function in prostate cancer patients - a prospective observational cohort analysis. BMC Cancer 12: 371. 22925152 10.1186/1471-2407-12-371 PMC3502584

[21] First MB, Spitzer RL, Williams JBW, Gibbon M (1995): Structured Clinical Interview for DSM-IV (SCID). American Psychiatric Association, Washington DC.

[22] Folstein MF, Folstein SE, McHugh PR (1975) “Mini-mental state”. A practical method for grading the cognitive state of patients for the clinician. J Psychiatr Res. 12: 189–198. 10.1016/0022-3956(75)90026-6 1202204

[23] Esper P, Mo F, Chodak G, Sinner M, Cella D, et al (1997) Measuring quality of life in men with prostate cancer using the functional assessment of cancer therapy-prostate instrument. Urology 50: 920–928. 9426724 10.1016/S0090-4295(97)00459-7

[24] Ashburner J, Friston KJ (2000) Voxel-based morphometry–the methods. Neuroimage 11: 805–821. 10860804 10.1006/nimg.2000.0582

[25] Manjon JV, Coupe P, Marti-Bonmati L, Collins DL, Robles M (2010) Adaptive non-local means denoising of MR images with spatially varying noise levels. J Magn Reson Imaging 31: 192–203. 20027588 10.1002/jmri.22003

[26] Rajapakse JC, Giedd JN, Rapoport JL (1997) Statistical approach to segmentation of single-channel cerebral MR images. IEEE Trans Med Imaging 16: 176–186. 9101327 10.1109/42.563663

[27] Tohka J, Zijdenbos A, Evans A (2004) Fast and robust parameter estimation for statistical partial volume models in brain MRI. Neuroimage 23: 84–97. 15325355 10.1016/j.neuroimage.2004.05.007

[28] Ashburner J (2007) A fast diffeomorphic image registration algorithm. Neuroimage 38: 95–113. 17761438 10.1016/j.neuroimage.2007.07.007

[29] Cherrier MM, Borghesani PR, Shelton AL, Higano CS (2010) Changes in neuronal activation patterns in response to androgen deprivation therapy: a pilot study. BMC Cancer 10: 1. 20047689 10.1186/1471-2407-10-1 PMC2824708

[30] Alibhai SM, Breunis H, Timilshina N, Marzouk S, Stewart D, et al (2010) Impact of androgen-deprivation therapy on cognitive function in men with nonmetastatic prostate cancer. J Clin Oncol. 28: 5030–7. 10.1200/JCO.2010.30.8742 21041708

[31] Witte AV, Savli M, Holik A, Kasper S, Lanzenberger R (2010) Regional sex differences in grey matter volume are associated with sex hormones in the young adult human brain. Neuroimage 49: 1205–12. 19796695 10.1016/j.neuroimage.2009.09.046

[32] Peper JS, Brouwer RM, Schnack HG, van Baal GC, van Leeuwen M, et al (2009) Sex steroids and brain structure in pubertal boys and girls. Psychoneuroendocrinology 34(3): 332–42. 18980810 10.1016/j.psyneuen.2008.09.012

[33] Neufang S, Specht K, Hausmann M, Güntürkün O, Herpertz-Dahlmann B, et al (2009) Sex differences and the impact of steroid hormones on the developing human brain. Cereb Cortex 19: 464–73. 18550597 10.1093/cercor/bhn100

[34] Bryant DM, Hoeft F, Lai S, Lackey J, Roeltgen D, et al (2011) Neuroanatomical phenotype of Klinefelter syndrome in childhood: a voxel-based morphometry study. J Neurosci. 31: 6654–60. 10.1523/JNEUROSCI.5899-10.2011 PMC3148194 21543594

[35] Itti E, Gaw Gonzalo IT, Pawlikowska-Haddal A, Boone KB, Mlikotic A, et al (2006) The structural brain correlates of cognitive deficits in adults with Klinefelter’s syndrome. J Clin Endocrinol Metab. 91: 1423–7. 10.1210/jc.2005-1596 16403821

[36] Mueller SC, Merke DP, Leschek EW, Fromm S, VanRyzin C, et al (2011) Increased medial temporal lobe and striatal grey-matter volume in a rare disorder of androgen excess: a voxel-based morphometry (VBM) study. Int J Neuropsychopharmacol. 14: 445–57. 10.1017/S1461145710001136 PMC4947374 20860880

[37] Azad N, Pitale S, Barnes WE, Friedman N (2003) Testosterone treatment enhances regional brain perfusion in hypogonadal men. J Clin Endocrinol Metab 88: 3064–3068. 12843144 10.1210/jc.2002-020632

[38] Forbes EE, Ryan ND, Phillips ML, Manuck SB, Worthman CM, et al (2010) Healthy adolescents' neural response to reward: associations with puberty, positive affect, and depressive symptoms. J Am Acad Child Adolesc Psychiatry 49: 162–72.e1–5. 20215938 10.1097/00004583-201002000-00010 PMC2837556

[39] Hermans EJ, Bos PA, Ossewaarde L, Ramsey NF, Fernández G, et al (2010) Effects of exogenous testosterone on the ventral striatal BOLD response during reward anticipation in healthy women. Neuroimage 52: 277–283. 20398773 10.1016/j.neuroimage.2010.04.019

[40] Moffat SD, Resnick SM (2007) Long-term measures of free testosterone predict regional cerebral blood flow patterns in elderly men. Neurobiol Aging 28: 914–920. 16698125 10.1016/j.neurobiolaging.2006.04.001

[41] van Wingen GA, Zylicz SA, Pieters S, Mattern C, Verkes RJ, et al (2009) Testosterone increases amygdala reactivity in middle-aged women to a young adulthood level. Neuropsychopharmacology 34: 539–547. 18235425 10.1038/npp.2008.2

[42] Mueller SC, Merke DP, Leschek EW, Fromm S, Grillon C, et al (2011) Grey matter volume correlates with virtual water maze task performance in boys with androgen excess. Neuroscience 197: 225–32. 21964472 10.1016/j.neuroscience.2011.09.022 PMC3210397

